# Correspondence: Spontaneous secondary mutations confound analysis of the essential two-component system WalKR in *Staphylococcus aureus*

**DOI:** 10.1038/ncomms14403

**Published:** 2017-02-06

**Authors:** Ian R. Monk, Benjamin P. Howden, Torsten Seemann, Timothy P. Stinear

**Affiliations:** 1 Department of Microbiology and Immunology, Peter Doherty Institute for Infection and Immunity, University of Melbourne, Melbourne, Victoria 3000, Australia; 2 Victorian Life Sciences Computation Initiative, University of Melbourne, Carlton, Victoria 3010, Australia

Ji *et al*.^1^ recently described the structure of the extracytoplasmic Per-Arnt-Sim (PAS) domain of WalK (WalK^EC-PAS^), the sensor kinase of the essential two-component system WalKR in *Staphylococcus aureus*. The authors made two independent *walK* mutants in *S. aureus*, each with a single amino acid alteration in WalK^EC-PAS^, inferring from the structure that these residues might be important for signal transduction. We have also been exploring the function of WalKR and were surprised by the striking phenotypic impacts of these single amino acid substitutions.

The authors showed that their WalK^EC-PAS^ mutants (WalK^D119A^ and WalK^V149A^) caused reduced susceptibility to lysostaphin, loss of sheep blood haemolysis, reduced biofilm formation and reduced virulence compared with parental methicillin-sensitive *S. aureus* strain Newman^2^. RNA-seq comparisons of the two mutants to wild type identified substantial transcriptional changes. Structure-based virtual screening was used to predict that 2,4-dihydroxybenzophenone (DHBP) would interact with WalK^EC-PAS^. As DHBP appeared to stimulate lysostaphin-induced lysis and biofilm formation in strain Newman, the authors postulated that it was activating WalKR. They then measured transcriptional responses of Newman after no treatment, DHBP exposure and in their D119A mutant and reported that there were 41 genes that inversely expressed the WalK^D119A^ mutant compared with DHBP-treated cells, concluding that this supported a role for DHBP in activating WalKR. No direct biochemical evidence of WalKR activation was presented^1^.

To investigate further, we were provided with *S. aureus* Newman wild-type, WalK^D119A^ and WalK^V149A^ by the senior author, Chuan He, University of Chicago (UoC)^1^. We sequenced the genomes of University of Melbourne (UoM) and UoC Newman strains and compared their sequences with the reference^2^, with the two strains differing by only one synonymous mutation in *sbnF* (NWMN_0065). Using allelic exchange, we recreated the WalK^D119A^ mutation in the Newman wild type and the USA300 lineage strain NRS384^3^. We confirmed by genome sequencing that only the AT→CG substitution in WalK^EC-PAS^ was introduced in both strains (NC_009641 chromosome position 25994). We then tested UoM Newman WalK^D119A^ and NRS384 WalK^D119A^ for the key phenotype changes observed by Ji *et al*.^1^. However, in contrast to Ji *et al*.,^1^ we observed that UoM WalK^D119A^ and NRS384 WalK^D119A^ were fully haemolytic (Fig. 1a) and exhibited identical growth curve kinetics as the parental strains (Fig. 1b). Concurrent screening of UoC Newman WalK^D119A^ confirmed that it was non-hemolytic (Fig. 1a). The mutant also grew to an increased OD_600_, as reported (Fig. 1b)^1^, although CFU were identical to wild type, suggesting that the increase in OD was not due to increased growth. Additionally, UoC WalK^D119A^ consistently exhibited larger colonies than Newman or UoM WalK^D119A^. We next measured the sensitivity of the strains to lysostaphin by cell viability (Fig. 1c). We observed that the UoC WalK^D119A^ mutant was significantly more sensitive (not resistant) to lysostaphin than wild type (3-log_10_ reduction versus Newman), whereas the UoM WalK^D119A^ mutant showed no change. Interestingly, the WalK^D119A^ mutation in NRS384 caused an increase in lysostaphin sensitivity, suggesting that the mutation contributes to WalKR activation rather than repression, as proposed by Ji *et al*.^1^,^4^.

To resolve the above discrepancies, we subjected UoC WalK^D119A^ and UoC WalK^V149^ to whole-genome sequencing. Relative to Newman, and in addition to their expected *walK*^EC-PAS^ changes, both UoC mutants D119A and V149A had acquired four additional mutations (Table 1). Most notably, two independent loss-of-function mutations in *saeRS*, a major two-component regulator that controls the expression of many genes involved in virulence and biofilm formation^5^,^6^,^7^,^8^. The UoC WalK^D119A^ had a TT insertion at position 757519 that introduced a frameshift to *saeS*. The UoC WalK^V149A^ had a G→T substitution at position 757889 that introduced a premature stop codon to *saeR*. It is these secondary mutations in *saeRS*, rather than the targeted mutations in *walK*^EC-PAS^, that likely explain the phenotypes observed by Ji *et al*.^1^ (reduced biofilm, loss of haemolysis, reduced virulence). We also mapped the authors' RNA-seq reads for WalK^D119A^ and WalK^V149A^ (GSE75731) and readily detected the same *saeRS* mutations. To confirm the predicted functional consequences of the *saeRS* mutations, we used a P1 Sae red fluorescence reporter plasmid^5^. No fluorescence activity was detected in the UoC WalK^D119A^ strain containing the P1 Sae reporter, consistent with the predicted truncation in the histidine kinase preventing phosphorylation of SaeR, whereas high-level expression of P1 Sae from Newman and UoM WalK^D119A^ was observed leading to red colonies (Fig. 1d). We then recreated the mutated *saeS* allele from UoC WalK^D119A^ in both wild-type Newman and UoM WalK^D119A^ (Fig. 1e). The mutation abolished haemolysis on sheep blood agar. We then repaired the *saeS* mutation in UoC WalK^D119A^ and observed restoration of wild-type haemolysis (Fig. 1e). These results show that the UoC WalK^D119A^ strain is an *sae* mutant with the majority of the phenotypic changes reported in this strain (including the reported RNA-seq changes) likely associated with this mutation rather than WalK^D119A^. The unintended secondary mutations in a major *S. aureus* regulatory locus preclude analysis of the role of WalK^EC-PAS^ domain in WalKR signal transduction.

There is precedence for this specific phenomenon. Sun *et al*.^5^ showed that elevated temperature and antibiotic selection used during the *S. aureus* mutagenesis process can aid in the selection of *saeRS* mutations. How Ji *et al*.^1^ managed to complement the mutations (D119A and V149A) by phage integrase plasmid expression (pCL55) of wild-type *walKR* remains to be explained. We have been unable thus far to obtain the complemented mutants for analysis.

We also observed that UoC WalK^D119A^ and UoC WalK^V149A^ exhibited larger colonies compared with wild type, a phenotypic difference not discussed by Ji *et al*.^1^. This change in both mutants might be explained by the C→T substitution observed at 820314, leading to an A128V change in HprK, a serine kinase known to be involved in catabolite repression and associated with a spreading colony phenotype^9^.

Ji *et al*.^1^ used RNA-seq and the inverse expression profiles of the WalK^D119A^ mutant and DHBP treatment of the wild type to ‘prove' that DHBP is signaling through WalK^EC-PAS^, but this conclusion is confounded by the *saeRS* mutations. In addition, the authors failed to apply any filter for false discovery rate to their RNA-seq analysis. This analysis without statistical significance thresholds is not meaningful^10^. We also repeated the lysostaphin assay with and without the addition of 75 μM DHBP. We failed to observe the reported loss of turbidity in Newman pretreated with DHBP upon lysostaphin treatment^1^ (Supplementary Fig. 1.).

The discovery of small-molecule inhibitors of WalKR function would represent a major advance in the fight against multidrug-resistant *S. aureus*. Unfortunately, the presence of unintended *saeRS* mutations in their *walK*^EC-PAS^ mutants invalidate their conclusions with respect to role of the WalK extracytoplasmic domain in controlling WalKR function. Using a clean D119A mutant, we observed opposing results: with increased sensitivity to lysostaphin, a phenotype previously linked with enhanced activity of WalKR^4^. In our own WalKR research, we have observed a propensity for mutations introduced into this locus to yield secondary compensatory events^11^. These secondary changes can confound analysis of this essential two-component system and highlight the extreme care needed when manipulating this locus and then attributing specific phenotypes to specific mutational changes.

## Methods

### Bacteria and molecular tools

The *S. aureus* UoM Newman was obtained from Professor Tim Foster (Trinity College Dublin); NRS384 was obtained from BEI resources (www.beiresources.org). *S. aureus* was routinely grown in Tryptic Soy Broth (TSB-Oxoid) at 37 °C with aeration at 200 r.p.m. Primers were purchased from IDT (www. idtdna.com) with primer sequences detailed in Supplementary Table 1. Restriction enzymes, Phusion DNA polymerase and T4 DNA ligase were purchased from New England Biolabs. Genomic DNA was isolated from 1 ml of an overnight culture (DNeasy Blood and Tissue Kit—Qiagen) pretreated with 100 μg of lysostaphin (Sigma cat. no. L7386). DHBP was purchased from Sigma (cat. no. 126217; 100 g).

### Lysostaphin sensitivity assay

Overnight cultures of *S. aureus* were diluted 1:100 in fresh, prewarmed TSB in the presence of 0.2 μg ml^−1^ of lysostaphin with or without 75 μM DHBP (100 mM stock in methanol). Broths were incubated statically at 37 °C. Colony-forming units were determined by spot plate dilution on Brain heart infusion agar (Difco) at 0 and 90 min. Limit of detection for the assay was 10^3^ CFU ml^−1^.

### Construction of pIMC8-RFP and SLiCE cloning

The *S. aureus* codon optimized DsRED red fluorescent protein and upstream TIR sequence from pRFP-F (ref. 12) was PCR amplified with primers IM314/IM315. The product was digested with KpnI/SacI and cloned into the complementary digested pIMC8 (non-temperature-sensitive version of pIMC5 (ref. 13)), creating pIMC8-RFP. To clone into pIMAY-Z^3^ and pIMC8-RFP, primers were tailed with 30 nt of complementary sequence to the plasmid. Amplimers were inserted with seamless ligation cloning extract (SLiCE; ref. 14) into the vector (pIMAY-Z: *walRK*^D119A^*, sae*^*STOP*^, *sae*^*FIX*^; pIMC8-RFP: P1 sae). Either vector was linearized with KpnI, gel extracted and PCR amplified with primers IM1/IM2 (pIMAY-Z) or IM1/IM385 (pIMC8-RFP). Both amplimers (vector and insert) were combined in a 10 μl reaction containing 1 × T4 ligase buffer, with 1 μl of SLiCE extract. The reaction was incubated at 37 °C for 1 h and then transformed into *Escherichia coli* strain IM08B^3^, with selection on Luria agar plates containing chloramphenicol 10 μg ml^−1^. Plasmids were extracted and directly transformed by electroporation into the target *S. aureus* strain^3^.

### Production of SLiCE extract

The SLiCE was isolated from DY380 (ref. 14) grown in 50 ml 2xYT (1.6% Tryptone, 1% Yeast Extract, 0.5% NaCl) at 30 °C after a 1:100 dilution of the overnight culture. Once the culture reached an OD_600_ of ∼2.5, the cells were moved to 42 °C for 25 min (addition of 50 ml of 42 °C 2xYT). Cells were processed as described by Zhang *et al*.^14^, with the pellet lysed in 500 μl of CelLytic B cell lysis reagent (C87040; 10 ml; Sigma).

### Whole-genome sequencing and data analysis

Whole-genome sequencing was performed using the Illumina NextSeq (2 × 150 bp chemistry), with library preparation using Nextera XT (Illumina). Resulting reads were mapped to the *S. aureus* Newman reference (Accession: NC_009641) using *Snippy* v3.1 (https://github.com/tseemann/snippy). Note that 89 substitutions, 20 deletions and 25 insertions were shared between UoC and UoM Newman strains compared with the NC_009641 reference sequence, representing likely sequencing errors in the 2008 published reference^2^.

### Data availability

All sequencing data used in this study have been deposited in the National Center for Biotechnology Information BioProject database and are accessible through the BioProject accession number PRJEB14381 (https://www.ncbi.nlm.nih.gov/bioproject/325902). An updated *S. aureus* Newman genome sequence is available (Genbank reference: NZ_LT598688.1).

## Additional information

**How to cite this article:** Monk, I. R. *et al*. Correspondence: Spontaneous secondary mutations confound analysis of the essential two component system WalKR in *Staphylococcus aureus*. *Nat. Commun*. **8,** 14403 doi: 10.1038/ncomms14403 (2017).

**Publisher's note**: Springer Nature remains neutral with regard to jurisdictional claims in published maps and institutional affiliations.

## Supplementary Material

Supplementary Information Supplementary Figure 1 and Table 1

## Figures and Tables

**Figure 1:**
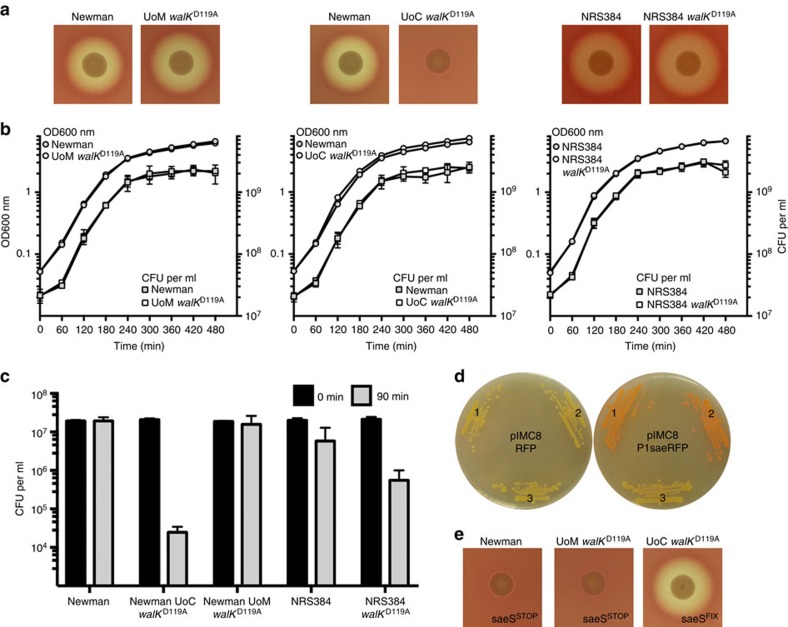
Phenotypic screening of *walK*^EC-PAS^ mutants. (**a**) No impact on haemolysis was observed for the WalK^D119A^ mutation in either Newman or NRS384 on sheep blood agar. (**b**) Growth kinetics were identical for the newly created WalK^D119A^ mutants in TSB at 37 °C with aeration (200 r.p.m.) when compared with the parental strain. Optical density (○) and colony-forming units (□) were enumerated. (**c**) In a lysostaphin growth sensitivity assay, the UoC WalK^D119A^ exhibited a loss of viability, while the parent or UoM WalK^D119A^ did not. The WalK^D119A^ mutation in the NRS384 background enhanced sensitivity to lysostaphin when compared with the parental strain. Error bars depict the s.d. of the mean from three independent experiments. (**d**) P1 sae promoter activity with a DsRED reporter. Left: no promoter. Right: P1 sae driving RFP expression; (1) Newman (2) UoM WalK^D119A^ (3) UoC WalK^D119A^. No expression of *sae* was observed in UoC WalK^D119A^. (**e**) By allelic exchange, the UoC *saeS* mutation was introduced into Newman or UoM WalK^D119A^ (*saeS*^STOP^) with haemolysis abolished, while introduction of the wild-type *saeS* gene into UoC WalK^D119A^ (*saeS*^FIX^) restored haemolysis.

**Table 1:** Whole-genome sequencing identified the introduction of unintended polymorphisms within the *S. aureus* Newman WalK mutants.

Position of *S. aureus* Newman (NC_009641)	UoC Newman (Acc: ERR1450026)	UoC Walk^D119A^ (Acc: ERR1450027)	UoC Walk^V149A^ (Acc: ERR1450028)	Locus_tag (NWMN_)	Gene	Comment (product, predicted consequence of mutation) c.=codon; p.=amino acid position
25994	A	C	A	0018	*walK*	Sensor kinase: missense_variant c.356A>C p.Asp119Ala
26084	T	T	C	0018	*walK*	Sensor kinase: missense_variant c.446T>C p.Val149Ala
87710	A	A	A	0065	*sbnF*	Siderophore biosynthesis: synomous c. 425G>A
757519	—	TT	—	0674	*saeS*	Sensor kinase: frameshift variant c.152_153ins AA p.Thr52fs
757889	C	C	A	0675	*saeR*	DNA-binding response regulator stop_gained c.469G>T p.Glu157*
820314	C	T	T	0728	*hprK*	HPr kinase/phosphorylase: missense_variant c.383C >T p.Ala128Val
897870	—	TG	TG	0810		Truncated hypothetical. Created full-length gene. c.98_99ins TG p. 50–119
1914999	T	T	C			Intragenic between NWMN_1716/1717
						
2370632	C	G	C	2142	*rplN*	50S ribosomal protein L14: missense_variant c.86G>C p.Gly29Ala
