# Supplementary material for: Correspondence: Spontaneous secondary mutations confound analysis of the essential two-component system WalKR in Staphylococcus aureus
Source: Nat Commun. 2017 Feb 6;8:14403. doi: 10.1038/ncomms14403 (PMC5303874; doi:10.1038/ncomms14403)
Supplement: Supplementary Information — Supplementary Figure 1 and Table 1 [file ncomms14403-s1.pdf]

Supplementary Information:

Supplementary Figure 1.

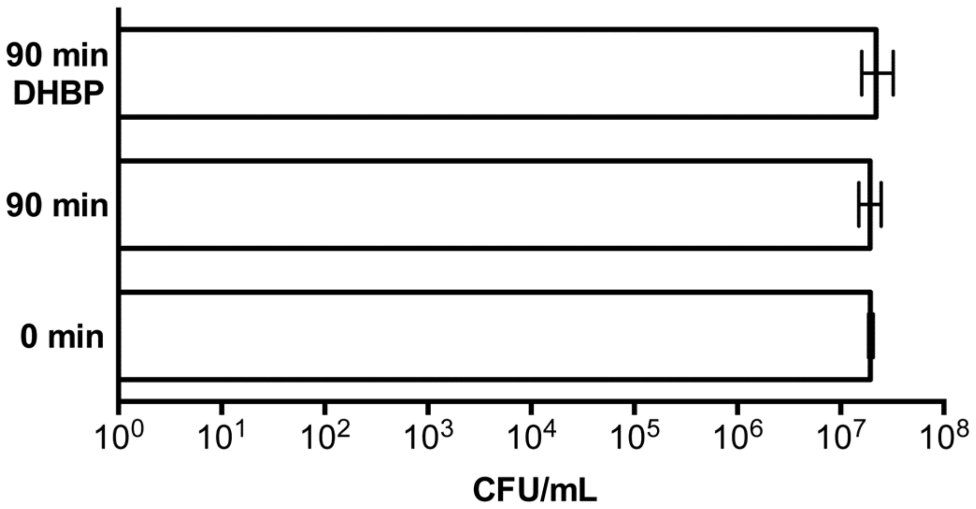

Supplementary Figure 1: Impact of DHBP on lysostaphin induced lysis. No enhanced impact on lysostaphin induced lysis in *S. aureus* strain Newman was observed when grown in the presence of 75 µM DHBP. Error bars depict the range from three independent experiments.

Supplementary Table 1: Oligonucleotides used in the study.

	**Oligonucleotide sequence (5'-3')**
**Allelic Exchange**	
**WalK D119A**	
IM107 walR AF	**CCTCACTAAAGGGAACAAAAGCTGGGTACC**ATGGCTAGAAAAGTTGTTGTAG
IM548 D119A walK BR	gcCATTCGCTTTTTGATTGATTAGACTACG
IM549 D119A walK CF	CCGTAGTCTAATCAATCAAAAAGCGAATGcgAGTTCTGTCCAAAAAGCACTATCAC
IM10 walK DR	**CGACTCACTATAGGGCGAATTGGAGCTC**CTCCTTATTATTCATCCCAATCACCGTC
IM552 D119A con F	CTAATCAATCAAAAAGCGAATGCG
IM1 pIMAY-Z F	GGTACCCAGCTTTTGTTCCCTTTAGTGAGG
IM2 pIMAY-Z R	GAGCTCCAATTCGCCCTATAGTGAGTCG
**SaeS**	
IM488 saeS AF	**CCTCACTAAAGGGAACAAAAGCTGGGTACC**TGATATCATGGTACTTGATATCATGATGC
IM488 saeS DR	**CGACTCACTATAGGGCGAATTGGAGCTC**TTGTAGAAGAAGTACAATTTGATGATGG
**P1sae reporter**	
IM494 P1 Sae F	**CCTCACTAAAGGGAACAAAAGCTGGGTACC**TTGGTACTTGTATTTAATCGTCTATC
IM495 P1 Sae R	**ATGTTTTTCCTCCTTATAAAGTTAATCA**TGTTGTGATAACAGCACCAGCTGC
IM314 TIR-RFP(KpnI) F	ATATGGTACCGGTGATTAACTTTATAAGGAGGAAAAACATATG
IM315 RFP(SacI) R	ATATGAGCTCAACATCTGTGGTATGGCGCTAGG
IM385 pIMC8-RFP R	TGATTAACTTTATAAGGAGGAAAAACATATG

Notes: Bold typeface: tails for recombination into the vector; Lower case: Mutation introduction; Underlined: Restriction site
